# Quantitative computed tomography determined regional lung mechanics in normal nonsmokers, normal smokers and metastatic sarcoma subjects

**DOI:** 10.1371/journal.pone.0179812

**Published:** 2017-07-27

**Authors:** Jiwoong Choi, Eric A. Hoffman, Ching-Long Lin, Mohammed M. Milhem, Jean Tessier, John D. Newell

**Affiliations:** 1 Departments of Radiology, University of Iowa, Iowa City, Iowa, United States of America; 2 IIHR-Hydroscience & Engineering, University of Iowa, Iowa City, Iowa, United States of America; 3 Departments of Biomedical Engineering, University of Iowa, Iowa City, Iowa, United States of America; 4 Departments of Medicine, University of Iowa, Iowa City, Iowa, United States of America; 5 Pharma Research and Early Development, Roche Innovation Center, Basel, Switzerland; Taipei Medical University, TAIWAN

## Abstract

**Objectives:**

Extra-thoracic tumors send out pilot cells that attach to the pulmonary endothelium. We hypothesized that this could alter regional lung mechanics (tissue stiffening or accumulation of fluid and inflammatory cells) through interactions with host cells. We explored this with serial inspiratory computed tomography (CT) and image matching to assess regional changes in lung expansion.

**Materials and methods:**

We retrospectively assessed 44 pairs of two serial CT scans on 21 sarcoma patients: 12 without lung metastases and 9 with lung metastases. For each subject, two or more serial inspiratory clinically-derived CT scans were retrospectively collected. Two research-derived control groups were included: 7 normal nonsmokers and 12 asymptomatic smokers with two inspiratory scans taken the same day or one year apart respectively. We performed image registration for local-to-local matching scans to baseline, and derived local expansion and density changes at an acinar scale. Welch two sample t test was used for comparison between groups. Statistical significance was determined with a p value < 0.05.

**Results:**

Lung regions of metastatic sarcoma patients (but not the normal control group) demonstrated an increased proportion of normalized lung expansion between the first and second CT. These hyper-expanded regions were associated with, but not limited to, visible metastatic lung lesions. Compared with the normal control group, the percent of increased normalized hyper-expanded lung in sarcoma subjects was significantly increased (p < 0.05). There was also evidence of increased lung “tissue” volume (non-air components) in the hyper-expanded regions of the cancer subjects relative to non-hyper-expanded regions. “Tissue” volume increase was present in the hyper-expanded regions of metastatic and non-metastatic sarcoma subjects. This putatively could represent regional inflammation related to the presence of tumor pilot cell-host related interactions.

**Conclusions:**

This new quantitative CT (QCT) method for linking serial acquired inspiratory CT images may provide a diagnostic and prognostic means to objectively characterize regional responses in the lung following oncological treatment and monitoring for lung metastases.

## Introduction

Human soft tissue sarcomas usually develop first in an extra thoracic location but have an increased propensity to metastasize to the lung. It has been noted that extra-thoracic tumors send out pilot cells that could attach to the pulmonary endothelium and could in theory alter regional lung mechanics by either stiffening the lung tissue or by increasing fluid and inflammatory cell accumulation near the pilot cell attachment in the endothelium producing changes in lung compliance [1–8]. We hypothesized in this study that prior to the computed tomographic-based (CT) visualization of a metastatic lung nodule, CT measures of regional lung mechanics [9–21] could detect changes in lung compliance not visible to the human eye on standard chest CT examinations and that these changes in compliance would precede the development of metastatic lung nodules that are subsequently visible on CT.

We designed a study to test this hypothesis by first developing a novel method to assess regional lung mechanics using non-rigid image registration methods that map one CT image study to another [9, 12, 16, 18]. In our application we choose two inspiratory thoracic CT scans both done at total lung capacity (TLC) but at two different time points. The earlier time point CT image data set was chosen to be the flexible image data set and the later time point CT was chosen to be the stationary or nonflexible image data set. Using this method we were able to look at small scale acinar level changes in regional lung volume between the two time points. This change in regional lung volume could be further broken down into air volume change and “tissue” volume change. Here the term “tissue” is used to refer to anything that is not air, including tissue, blood, extravascular lung water, inflammatory cells or intravenously injected intra- and extra-vascular iodinated contrast media.

We demonstrate that at TLC there are regions of the lungs of sarcoma subjects which become increasingly hyper-expanded relative to the remainder of their lung and some of these hyper-expanded regions associate with the appearance of metastatic lesions, and the hyper-expanded regions, along with their increased air content, are found to have increased tissue (including blood) content. As a validation step, we assessed a control group of nonsmokers that showed no signs of regional hyper-expansion upon repeat imaging.

## Materials and methods

### Subject groups

Three groups of subjects were recruited into our study. Group 1 consisted of 7 normal nonsmokers. Each of these subjects had already undergone thoracic CT scanning as part of an NIH Biomedical Research Partnership grant (HL-064368) designed to construct an atlas of the normal human lung. These subjects varied in age from 21 to 43 years, 2 males and 5 females. Each of these subjects had two sequential CT scans performed with lung volume held and volume controlled at TLC, 30 minutes apart with the subject getting off and back onto the scanner table between scans. Group 2 was made up of 12 normal smoking subjects who were previously studied at the University of Iowa as part of another IRB approved multicenter study and had two CT scans each performed with lung volume held at a verbally coached TLC one year apart. These subjects varied in age from 40 to 73 years, 7 males and 5 females, and had smoking history of 32±20 pack-years. Group 2 subjects were included for comparison of long term changes since the two scans have a much longer time interval (one year) than Group 1. Group 3 subjects included 21 sarcoma subjects from the Melanoma and Sarcoma Tissue and Epidemiology Resource (MAST) program in the University of Iowa Holden Comprehensive Cancer Center that each had a tissue diagnosis of malignant extra-thoracic sarcoma. 12 of these sarcoma subjects did not have visible evidence of lung metastases on the follow up chest CT and 9 of these sarcoma subjects developed visible lung metastases on the follow up chest CT study. These subjects varied in age from 21 to 73 years, 12 males and 9 females. 9 were nonsmokers, 6 smokers had smoking history of 44±21 pack-years, and smoking information were unavailable for 6 subjects. Each of the sarcoma subjects had two or more sequential clinical CT scans done while asked to hold their breath at a full inspiration. Scanning was at intervals that varied from 2 to 25 months. A total 65 clinical scans were done on a number of different CT scanners with several clinical CT protocols including many that had iodinated intravenous contrast media administered prior to the CT scan. A summary of the three groups in the study are provided in **Table 1**.

**Table 1:** Human subjects: Two control groups and sarcoma subjects.

Category	Group	Scan Interval	No. Subjects	%Total
Control (Normal nonsmokers)	Group 1	Same day	7	17.5%
Control (Asymptomatic smokers)	Group 2	1 year	12	30.0%
Cancer (sarcoma) subjects	Group 3	2–25 Months	21	52.5%
Sarcoma (No lung metastases)	Group 3	2–25 Months	12	30.0%
Sarcoma (Lung metastases)	Group 3	2–25 Months	9	22.5%
Total subjects all categories			40	100%

### CT scan protocols

Group 1 normal nonsmoking subjects were scanned on Siemens Sensation 64-slice MDCT scanner (Forchheim, Germany). Scan type, peak voltage, effective current and slice thickness were selected as spiral, 120 kV, 75 mAs and 0.75 mm, respectively. Each volumetric data set was acquired at a slice spacing of 0.5 mm and a reconstruction matrix of 512 × 512 using the B35f kernel. Group 2 normal smoking subjects were scanned as part of another multi-center study on a variety of scanners utilizing the previously published SPIROMICS scan protocol [22]. Slice thickness, slice spacing for volumetric reconstruction, and reconstruction matrix dimensions were 0.625–0.75 mm, 0.5 mm, and 512 × 512, respectively. Group 3 extra-thoracic sarcoma subjects had their CT scans performed on various CT scanners and various protocols were used for volumetric reconstruction. We have summarized this in **Table 2**.

**Table 2:** CT scan information of Group 3 sarcoma subjects.

Subject	Scan	Interval (months)	Slice Thickness	Voxel Dimensions (x, y, z)			Scanner		Kernel
1	1		2	0.7500	0.7500	1	Siemens	Sensation 16	B31f
1	2	3	2	0.7813	0.7813	1	Siemens	Sensation 16	B31f
1	3	7	2	0.7813	0.7813	1	Siemens	Sensation 16	B31f
2	1		2	0.6406	0.6406	1	Siemens	Definition	B20f
2	2	15	3	0.5684	0.5684	3	Siemens	Definition AS	B31f
2	3	25	3	0.6348	0.6348	3	Siemens	Definition AS	I31f_2
3	1		2	0.7695	0.7695	1	Siemens	Definition AS+	B70f
3	2	12	3	0.8203	0.8203	1.5	Siemens	Sensation 16	B31f
3	3	25	3	0.7305	0.7305	1.5	Siemens	Definition AS	B31f
3	4	12	3	0.7734	0.7734	1.5	Siemens	Definition AS	B31f
4	1		2	0.7188	0.7188	1	Siemens	Sensation 16	B70f
4	2	4	2	0.6211	0.6211	1	Siemens	Definition AS	B70f
4	3	9	2	0.6777	0.6777	1	Siemens	Sensation 16	B70f
5	1		3	0.7227	0.7227	1.5	Siemens	Definition AS	B31f
5	2	4	2	0.7402	0.7402	1	Siemens	Definition AS	I70f_2
5	3	2	2	0.7402	0.7402	1	Siemens	Definition AS	B70f
5	4	6	2	0.7559	0.7559	1	Siemens	Definition AS	B70f
6	1		2	0.7422	0.7422	1	Siemens	Sensation 16	B31f
6	2	4	2	0.6426	0.6426	1	Siemens	Definition AS	I70f_2
7	1		2	0.6621	0.6621	1	Siemens	Sensation 16	B31f
7	2	3	2	0.6680	0.6680	2	Siemens	Definition AS	B70f
7	3	22	2	0.6641	0.6641	1	Siemens	Definition AS	B70f
8	1		3	0.6719	0.6719	1.5	Siemens	Sensation 64	B20f
8	2	3	2	0.6445	0.6445	1	Siemens	Sensation 16	B70f
8	3	4	2	0.6211	0.6211	1	Siemens	Definition AS	B70f
8	4	3	2	0.6563	0.6563	2	Siemens	Definition AS	B70f
8	5	1	2	0.6816	0.6816	1	Siemens	Definition AS	B70f
9	1		2	0.6680	0.6680	1	Siemens	Definition AS	I70f
9	2	4	2	0.6992	0.6992	1	Siemens	Definition	B70f
10	1		2	0.8340	0.8340	1	Siemens	Definition AS	B70f
10	2	10	2	0.9141	0.9141	1	Siemens	Sensation 16	B70f
11	1		5	0.6641	0.6641	2.5	GE	Lightspeed VCT	Standard
11	2	4	2	0.6289	0.6289	1	Siemens	Definition AS	B70f
12	1		3	0.6641	0.6641	1.5	Siemens	Sensation 16	B31f
12	2	20	3	0.6738	0.6738	1.5	Siemens	Sensation 16	B20f
13	1		2	0.6777	0.6777	1	Siemens	Sensation 16	B70f
13	2	2	2	0.6660	0.6660	1	Siemens	Sensation 16	B70f
14	1		2	0.7168	0.7168	1	Siemens	Sensation 16	B70f
14	2	3	2	0.6953	0.6953	1	Siemens	Sensation 16	B70f
14	3	3	2	0.7324	0.7324	2	Siemens	Definition AS	B70f
15	1		3	0.7969	0.7969	1.5	Siemens	Sensation 64	B20f
15	2	11	2	0.7969	0.7969	1	Siemens	Definition	B20f
15	3	1	2	0.7461	0.7461	1	Siemens	Sensation 16	B70f
15	4	2	2	0.7285	0.7285	1	Siemens	Definition	B70f
16	1		2	0.9141	0.9141	1	Siemens	Sensation 16	B31f
16	2	1	2	0.8203	0.8203	1	Siemens	Sensation 16	B70f
16	3	3	3	0.9492	0.9492	1.5	Siemens	Definition AS	B31f
16	4	3	2	0.8320	0.8320	1	Siemens	Definition	B20f
17	1		2	0.6758	0.6758	1	Siemens	Sensation 16	B70f
17	2	3	2	0.6719	0.6719	1	Siemens	Sensation 64	B70f
17	3	3	2	0.6953	0.6953	1	Siemens	Sensation 16	B70f
18	1		3	0.9336	0.9336	1.5	Siemens	Definition AS	B31f
18	2	1	2	0.7715	0.7715	1	Siemens	Definition	B31f
18	3	2	2	0.791	0.791	1	Siemens	Sensation 16	B31f
19	1		2	0.9512	0.9512	1	Siemens	Sensation 16	B31f
19	2	2	2	0.8066	0.8066	1	Siemens	Definition AS	B70f
19	3	6	3	0.8867	0.8867	1.5	Siemens	Definition AS	I31f_2
19	4	3	3	0.9277	0.9277	1.5	Siemens	Definition AS	B31f
20	1		2	0.7207	0.7207	1	Siemens	Sensation 16	B70f
20	2	3	3	0.7812	0.7812	1.5	Siemens	Sensation 16	B31f
20	3	4	2	0.75	0.75	1	Siemens	Sensation 16	B31f
20	4	2	2	0.7266	0.7266	2	Siemens	Sensation 16	B31f
21	1		2	0.6543	0.6543	1	Siemens	Sensation 64	B70f
21	2	2	2	0.6816	0.6816	1	Siemens	Sensation 16	B70f
21	3	22	2	0.6777	0.6777	1	Siemens	Sensation 16	B70f

### CT image segmentation

**Fig 1:**
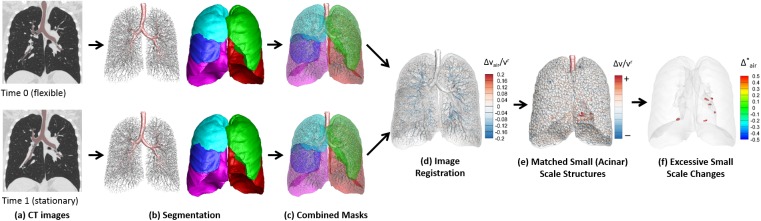
Schematic of flow chart to assess regional lung mechanics based on a pair of serial CT scans.

**Fig 1** shows the flow chart to assess regional lung mechanics based on two temporally sequenced images. For a pair of full inspiration volumetric CT image data sets obtained at different times (Time 0 and Time 1), each data set was segmented for the airway, vessels, lungs, and lobes, utilizing the Apollo 2.0 workstation (VIDA Diagnostics, Coralville, Iowa).

### CT image registration

The mass preserving non-rigid image registration method [16] was employed to obtain local-to-local matching of volumetric CT images from Time 0 to Time 1 using the combined masks of segmented geometries listed above. The CT images at Time 0 and Time 1 are used for the floating and the reference images, respectively. In a voxel, the tissue volume is calculated by:

$$v_{tissue}(x)=v(x)\frac{I(x)-{HU}_{air}}{{HU}_{tissue}-{HU}_{air}}$$ (1)

*v*_*tissue*_(***x***), *v*(***x***), *I*(***x***), *HU*_*air*_, and *HU*_*tissue*_ denote tissue volume, voxel volume, CT intensity of a voxel, Hounsfield unit of air, and Hounsfield unit of tissue, respectively. The bounds of *I*(***x***) are set with the Hounsfield units of -1000 and 55 for air and tissue, respectively [16]. The method determines a spatial transformation that matches the two images by minimizing a cost function that is the sum of squared tissue volume difference (SSTVD) as defined as:

$$E=\sum_{x\in \Omega }{\left(v_{tissue}^{r}(x)-v_{tissue}^{f}(T(x))\right)}^{2},$$ (2)

$v_{tissue}^{r}$ and $v_{tissue}^{f}$ are the tissue volumes of the reference and floating images, respectively. *T*(***x***) is the warping function, which is optimized to map the voxel location ***x*** in the reference image to the floating image. Once the tissue volume is known from Eq (1), the air volume can be calculated by *v*_*air*_ = *v* − *v*_*tissue*_ at *x* and *T*(*x*) in the reference image and the floating image[16]. The cost function serves to minimize the local tissue volume difference within the lungs between matched regions, preserving the tissue mass of the lungs if the tissue density is assumed to be constant in the lung.

### Regional lung mechanics calculations

**Fig 2:**
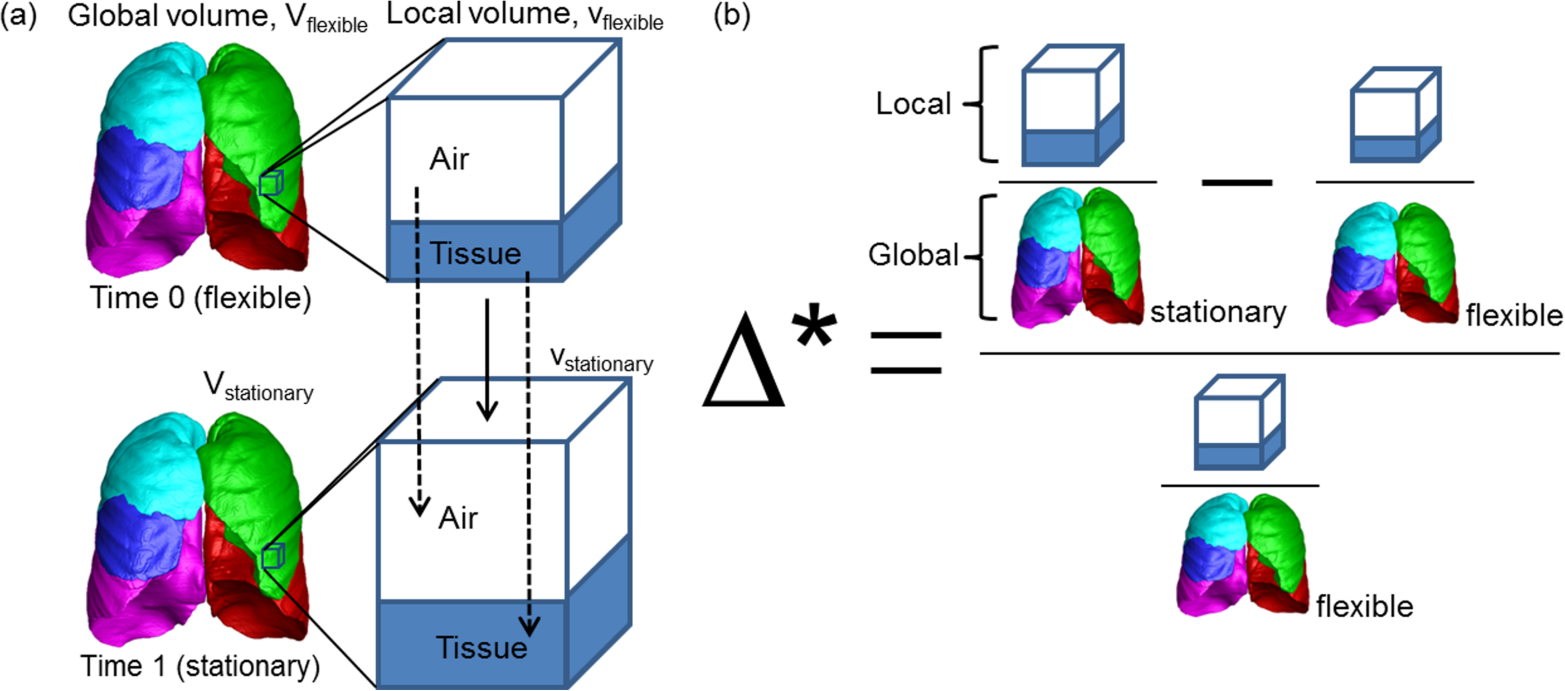
Schematic of (a) global and local lung volume changes and (b) formulation of normalized fractional volume change.

From matched local points, we compute the changes of biomechanical variables between two scans. In order to minimize the influences of variable global lung volumes or scan parameters between different scans of the same subjects or between different subjects on the assessment of biomechanical changes, we propose a normalized local variable to measure changes in geometrical volume (Δ*), air volume ($\Delta_{air}^{*}$), and tissue volume ($\Delta_{tissue}^{*}$). **Fig 2** shows the schematic of (Δ*). We first compute the local contribution to the global lung volume, and calculate the normalized change with respect to the value at the earlier time point, Time 0, as shown in Eqs (3), (4) and (5) for Δ*, $\Delta_{air}^{*}$, and $\Delta_{tissue}^{*}$, respectively.

$$\Delta^{*}=\frac{\Delta (\frac{v}{V})}{{\left(\frac{v}{V}\right)}^{f}}=\frac{\frac{v^{r}}{V^{r}}-\frac{v^{f}}{V^{f}}}{\frac{v^{f}}{V^{f}}}=\frac{\frac{v^{r}}{V^{r}}}{\frac{v^{f}}{V^{f}}}-1=\frac{v^{r}}{v^{f}}\frac{V^{f}}{V^{r}}-1=J\frac{V^{f}}{V^{r}}-1$$ (3)

where *V* is the total (geometrical) volume of the global lung, and superscripts *r* and *f* denote reference and floating images. Local Jacobian determinant *J* is the volume fraction of the matched voxel volumes, that is, *J* = *v*^*r*^/*v*^*f*^. Thus, Δ* is the proposed fractional volume change, which implies the change in local to global volumetric contribution normalized by that of the image at the earlier time point Time 0, and is used to measure local expansion between two time points. If Δ* is 0.5, for example, the local volumetric contribution of the region to the global lung has increased by 50% from Time 0 to Time 1. Decomposing a voxel volume into two components, air and tissue, based on CT density, “tissue” does not merely mean the soft tissue component but includes all non-air components such as blood, inflammatory cells with associated fluid, and any intravenously administered intravascular or extravascular iodinated contrast media.

Similarly to the fractional total (geometrical) volume, Δ*, fractional air volume change $\Delta_{air}^{*}$ is computed by Eq (4).

$$\Delta_{air}^{*}=\frac{\Delta (\frac{v_{air}}{V})}{{\left(\frac{v}{V}\right)}^{f}}=\frac{\frac{v_{air}^{r}}{V^{r}}-\frac{v_{air}^{f}}{V^{f}}}{\frac{v_{air}^{f}}{V^{f}}}=\frac{\frac{v_{air}^{r}}{V^{r}}}{\frac{v_{air}^{f}}{V^{f}}}-1=\frac{v_{air}^{r}}{v_{air}^{f}}\frac{V^{f}}{V^{r}}-1$$ (4)

Fractional tissue volume change is computed by Eq (5).

$$\Delta_{tissue}^{*}=\frac{\Delta (\frac{v_{tissue}}{V})}{{\left(\frac{v}{V}\right)}^{f}}=\frac{\frac{v_{tissue}^{r}}{V^{r}}-\frac{v_{tissue}^{f}}{V^{f}}}{\frac{v_{tissue}^{f}}{V^{f}}}=\frac{\frac{v_{tissue}^{r}}{V^{r}}}{\frac{v_{tissue}^{f}}{V^{f}}}-1=\frac{v_{tissue}^{r}}{v_{tissue}^{f}}\frac{V^{f}}{V^{r}}-1$$ (5)

### Statistical analysis

Welch two sample t tests were used to compare the control subjects with normal smokers and sarcoma subjects. The Welch t-test can help compensate for the different variance in each of the 3 groups. Statistical significance was achieved if p < 0.05.

## Results

### Normalized fractional volume change and tissue change

The regional mechanical index for total volume, $\Delta_{50\%}^{*}$, which denotes the percent lung volume represented by the regions where the normalized volume change is equal to or greater than 50%, was 0.056±0.079% in normal nonsmokers (Group 1) indicating no significant hyper-expanded regions. The lung regions where the normalized change in tissue volume is equal to or greater than 50%, $\Delta_{tissue,50\%}^{*}$, was 4.13±1.2%. There was a significant increase in $\Delta_{50\%}^{*}$ by 0.208%±0.22% (p < 0.0406) in the normal smoking group (Group 2) compared to the normal control group (Group 1) likely related to subclinical small airway disease. There was no significant increase in $\Delta_{tissue,50\%}^{*}$ when comparing Group 1 subjects to Group 2 subjects, (p > 0.05). Group 3 sarcoma subjects without lung metastases had significant increases in $\Delta_{50\%}^{*}$ when compared to Group 1 subjects, 4.5±6.5% (p < 0.001), and $\Delta_{tissue,50\%}^{*}$, 15.4±(13%) (p < 0.0001). Group 3 sarcoma subjects with lung metastases had significant increases in $\Delta_{50\%}^{*}$ compared to the Group 1 subjects, 1.7%±2.2% (p < 0.03), and near significant increases in $\Delta_{tissue,50\%}^{*}$ compared to the Group 1 subjects, 8.0±6.8% (p = 0.08). The results of the regional lung mechanical differences between groups are summarized in **Table 3**. For Group 3 subjects, percent of the lung volume representing hyper-expanded regions varied between pairs of serial CT scans. 33 (19+14), 25 (14+11), 23 (13+10), 19 (12+7) pairs out of total 44 (23+21) pairs from 21 (12+9) subjects had hyperinflation applying thresholds that 0.1%, 0.5%, 1%, and 2% lung volume have hyper-expanded based on $\Delta_{50\%}^{*}$.

**Table 3:** Percent lung volume of hyper-expanded regions and regions of expanded tissue volume.

Group	Number (CT pairs)	%Lung with Δ* ≥ 50%, mean (SD), p-value*: Hyper-expanded regions	%Lung with Δ*_tissue_ ≥ 50%, mean (SD), p-value: Regions with expanded “tissue” volume
Group 1	7	0. 056% (0.079%)	4.13% (0.93%)
Group 2	12	0.208% (0.22%), p < 0.046	5.3% (4.0%), p > 0.05 (p = 0.3686)
Group 3 (No lung metastases)	23	4.5% (6.5%), p < 0.001	15.4% (13%), p < 0.0001
Group 3 (Lung metastases)	21	1.7% (2.2%), p < 0.03	8.0% (6.8%), p = 0.08

^*^p value: Welch two sample t test relative to normal control

**Fig 3:**
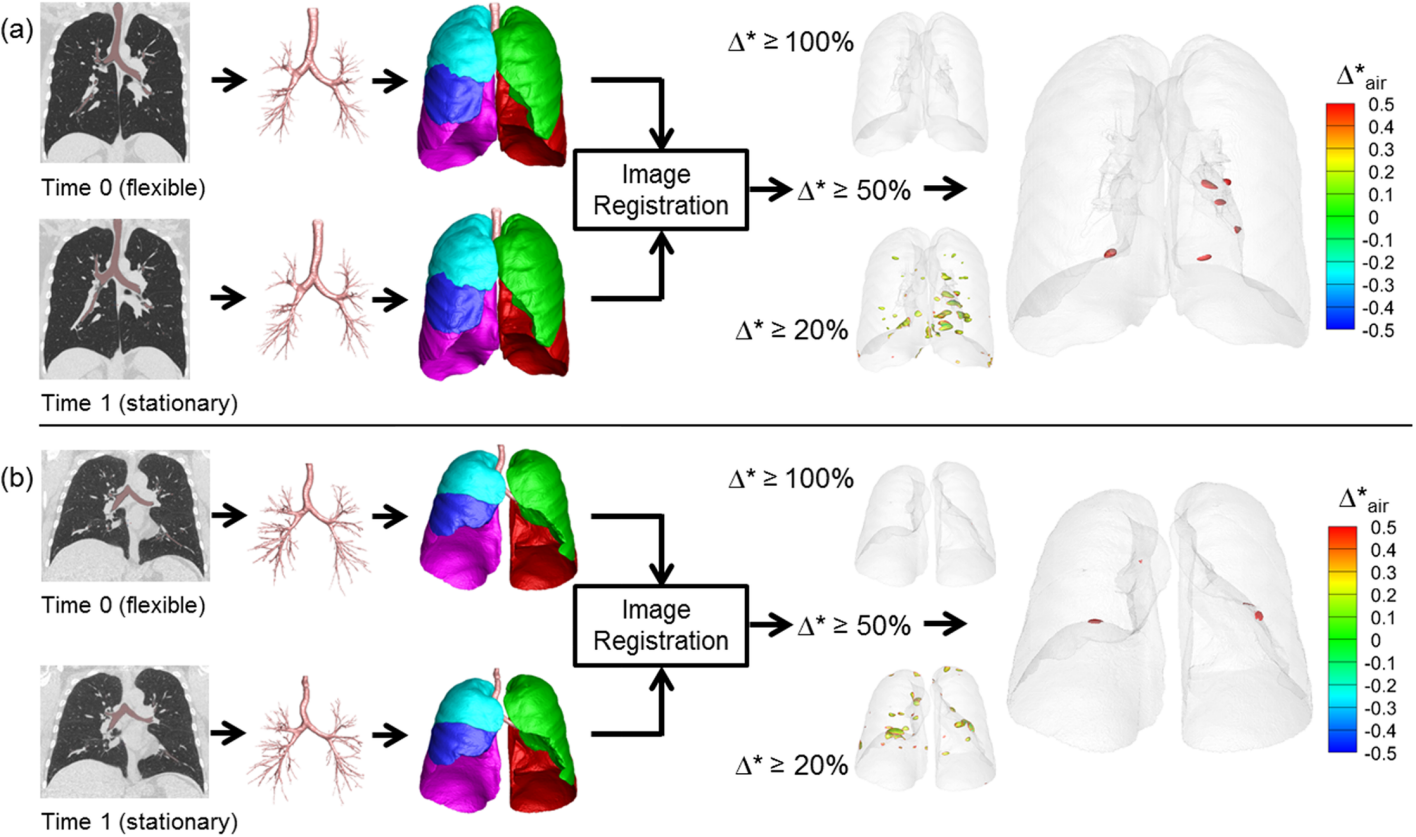
Regional hyper-expansion signals in serial CT pairs of two control subjects. (a) Normal non-smoker (Group 1) and (b) Normal smoker (Group 2).

**Fig 4:**
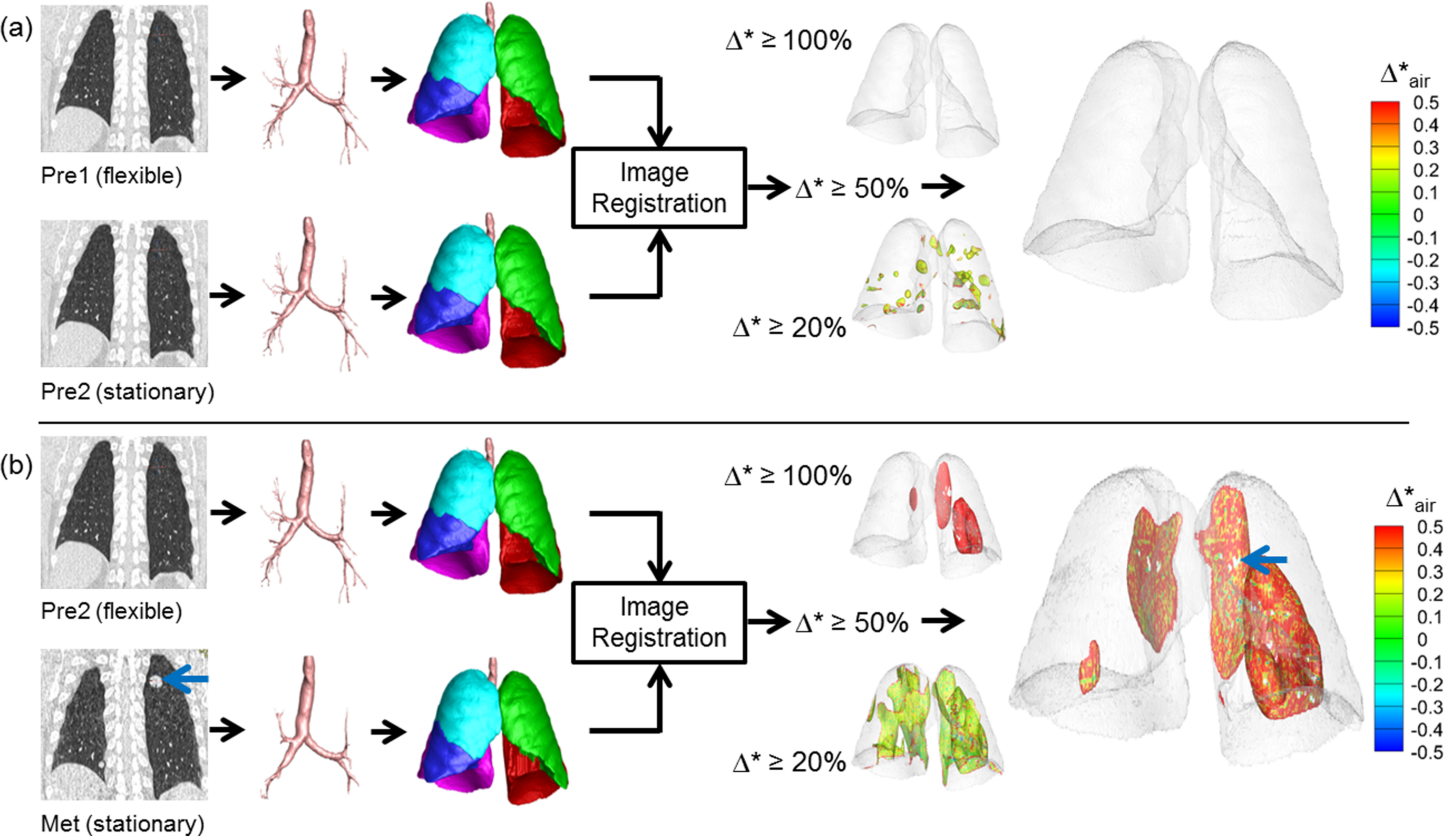
Regional hyper-expansion signals in serial CT pairs of a sarcoma patient (Group 3) with lung metastasis. (a) Pre1 and Pre2 and (b) Pre2 to Met. Lung metastases were not found at two earlier time points Pre1 and Pre2. A large metastatic nodule was noted on the 3rd scan, Met, indicated by the blue arrow.

**Fig 5:**
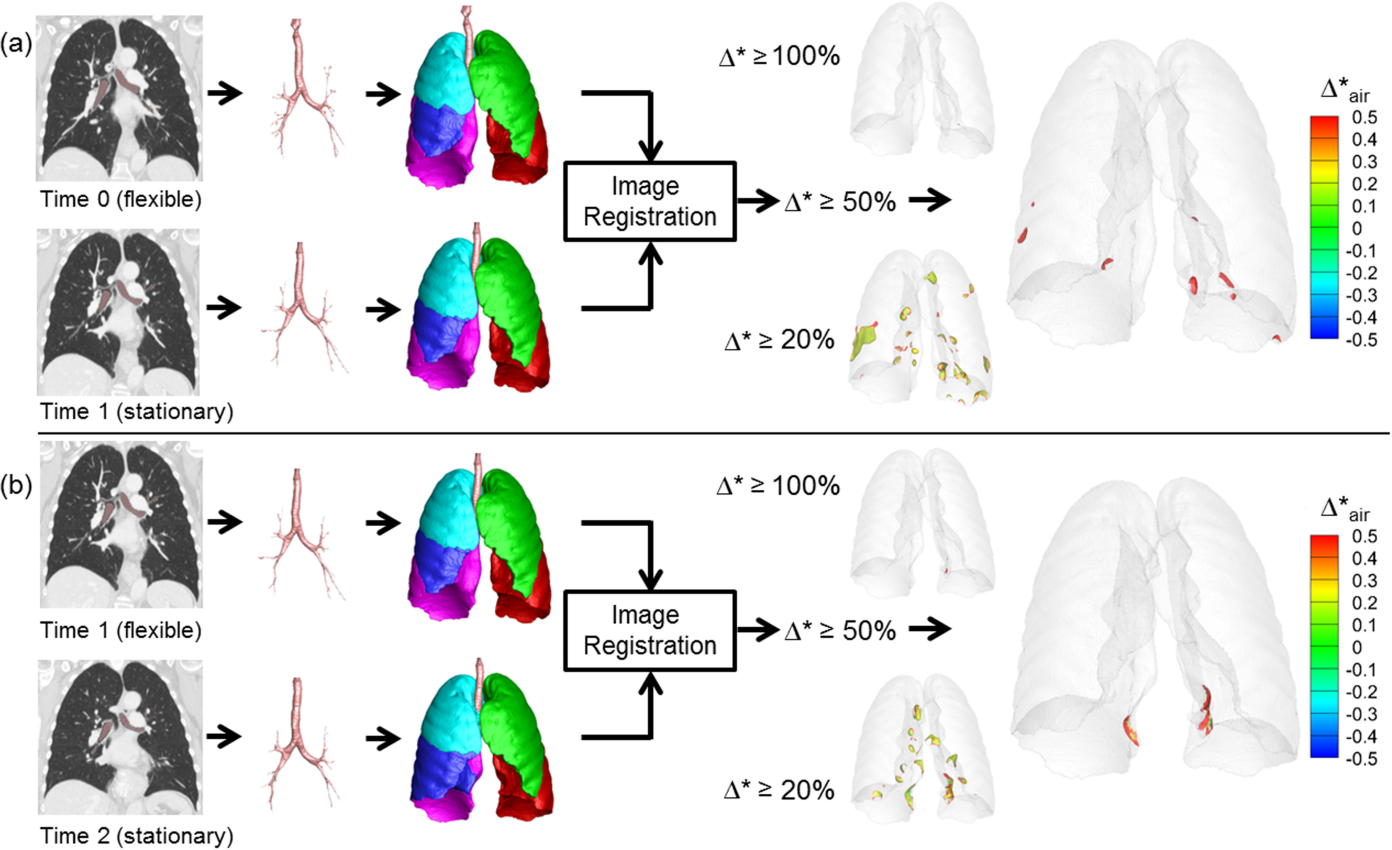
Regional hyper-expansion signals in serial CT pairs of a sarcoma patient (Group 3) with no lung metastasis and no therapy or resection. (a) Time 0 to Time 1 and (b) Time 1 to Time 2. Lung metastases were not found at any of three time points, Time 0, Time 1 and Time 2.

**Fig 6:**
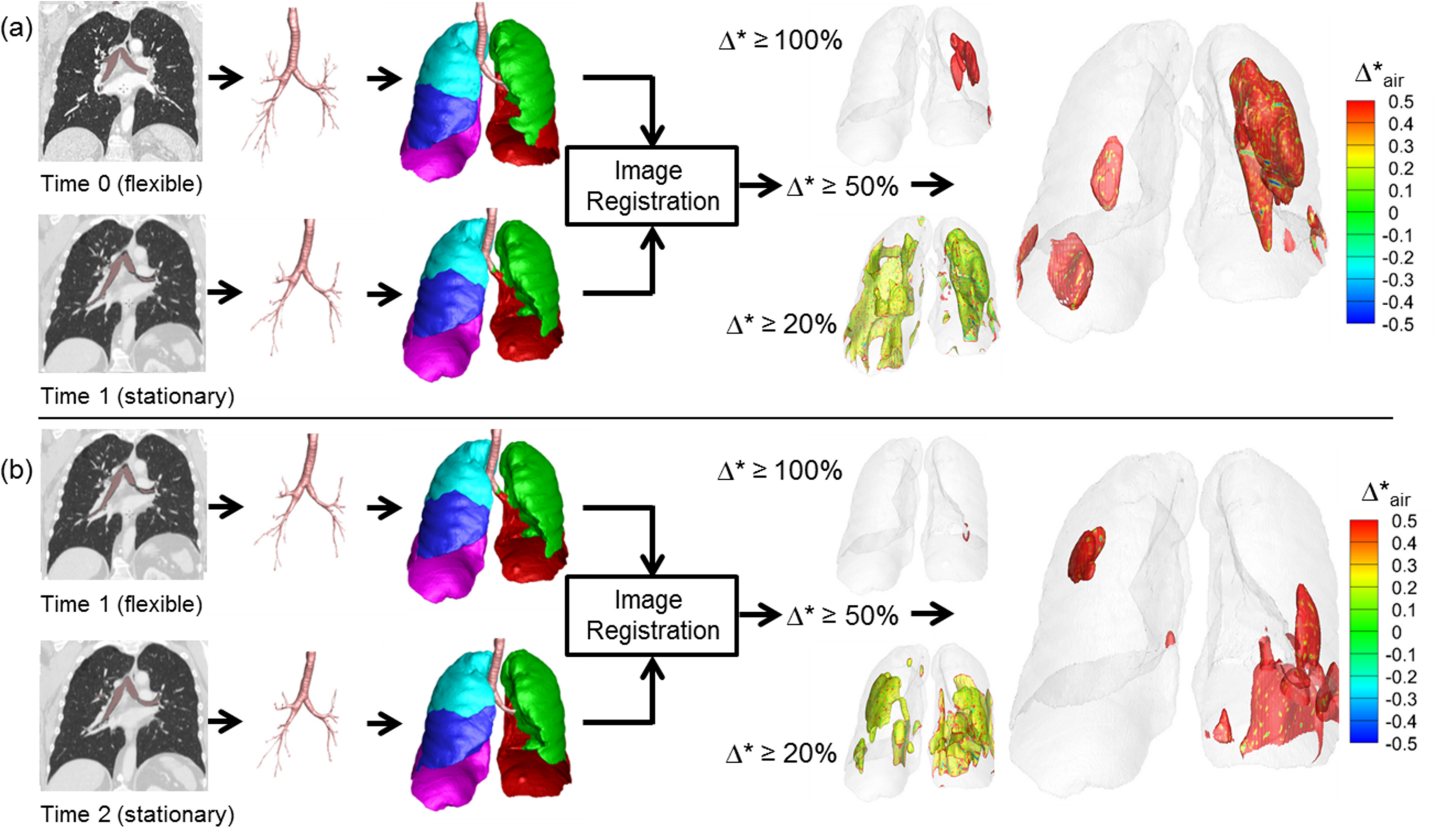
Regional hyper-expansion signals in serial CT pairs of a sarcoma patient (Group 3) with no lung metastasis but with therapy and resection. (a) Time 0 to Time 1 and (b) Time 1 to Time 2. Lung metastases were not found at any of the three time points, Time 0, Time 1 and Time 2.

**Fig 3** shows representative results of Group 1 control subjects. Segmented airways, lungs and lobes are nearly identical between Time 0 and Time 1. Using thresholds of 20%, 50%, and 100%, regions of normalized fractional volume change, Δ*, are presented. The hyper-expanded regions using a given threshold are color-coded by the amount of increase or decrease in air content in the hyper-expanded region. Of note is the fact that in all of the hyper-expanded regions determined by our image matching method, there is also an increase in air volume. Both Group 1 and Group 2 subjects show only minimal regions of normalized hyper-inflation. Small scattered regions are observed using a 20% threshold and these regions are reduced when the threshold is increased to 50%. Group 1 results were generated using two same day TLC chest CT scans demonstrating that local contribution to the global volume and its change are repeatable. Group 2 subjects with 1 year time intervals also show minimal hyperinflation similar to Group 1. They have slightly greater variability between subjects, which could be due to the long interval between scans or smoking-induced changes in the lung. This group was also scanned in the context of a multi-center research study and not in a single research dedicated scanning facility used for Group 1 subjects. **Fig 4** shows a representative subject of Group 3, who has two pre-metastatic scans and a post-metastatic scan. The hyperinflation between the two pre-metastatic scans (Pre1 and Pre2) show only minimal increases compared to the control subjects for thresholds of 20%, 50%, and 100%. In the third scan (Met), however, a metastatic lung nodule has grown in the left upper lobe denoted an arrow. From Pre2 to Met scans, larger regions of hyper-expansion were found using each of the 20%, 50%, and 100% thresholds. These findings were noted in the regions surroundings the cancer nodule and also in regions distant to the lung nodule including the contralateral lung. **Fig 5** presents a Group 3 subject with three non-metastatic scans. Hyper-expanded regions were minimal as in normal subjects. Time 0 to Time 1 interval is 3 months and Time 1 to Time 2 interval is 8 months, and the subject had no treatment or resection between Time 0 and Time 2. The subject shown in **Fig 6** is another Group 3 subject without visible metastatic lung lesions on CT. However, considerable regions of hyperinflation were found using a thresh hold of 20% and 50%. Time 0 to Time 1 interval is about 1 year and Time 1 to Time 2 interval is about 2 years, and the subject received drug therapy and surgical resections of the primary tumor between CT scans.

Hyperinflation of the scan pairs of sarcoma patients with lung metastasis were visually evaluated as presented in **Table 4**. Among Group 3 subjects with lung metastases, 11 of 21 pairs are metastatic pairs. Pre to Met pairs and Met to Met pairs are all regarded as metastatic pairs. All 11 metastatic pairs show hyperinflation based on the $\Delta_{50\%}^{*}$ criteria. 2 of 10 non-metastatic pairs (pair 8 and 10 in **Table 4**, respectively) also show hyperinflation, which may be a precursor of pre-metastatic niche in the lung with inflammatory process.

**Table 4:** Hyperinflation from visual inspection of sarcoma subject pairs with metastases.

k^*^	Subject	Time0	Time1	Hyperinflation	*k*	Subject	Time0	Time1	Hyperinflation
1	13	Pre	Met	o	12	18	Pre1	Pre2	x
2	14	Pre1	Pre2	x	13	18	Pre2	Met	o
3	14	Pre2	Met	o	14	19	Pre1	Pre2	x
4	15	Pre1	Pre2	x	15	19	Pre2	Met1	o
5	15	Pre2	Met1	o	16	19	Met1	Met2	o
6	15	Met1	Met2	o	17	20	Pre1	Pre2	x
7	16	Pre1	Pre2	x	18	20	Pre2	Pre3	x
8	16	Pre2	Pre3	o	19	20	Pre3	Met	o
9	16	Pre3	Met	o	20	21	Met1	Met2	x
10	17	Pre1	Pre2	o	21	21	Met2	Met3	o
11	17	Pre2	Met	o					

* *k* indicates the pair number.

## Discussion

### Alteration in regional lung mechanics

The results of our study support our hypothesis that regional lung mechanics are altered in subjects with extra thoracic soft tissue sarcomas and regional lung mechanics are not significantly altered in subjects without sarcomas when studied within the same day or across a 1 year interval. Regional lung mechanics were altered in sarcoma subjects that developed CT visible lung metastases and also in sarcoma subjects that, at least during the time interval of our study, had not developed lung metastases. There were no sarcoma subjects that developed CT visible lung metastases with normal QCT measures of regional lung mechanics. For QCT measures of changing regional lung mechanics in patients who did not have visible lung metastasis, we hypothesize that these changes might be attributed to other inflammatory driven effects including unsuccessful proliferation of pilot cells due a successful host response to destroy tumor cells, chemotherapy induced inflammation in the lung or other inflammatory injuries such as aspiration or infection.

The clinical importance of early detection of lung metastases and their effect on patient outcomes is very significant [23–25]. For this reason, we believe that patients with peripheral extra-thoracic soft tissue sarcomas can benefit from our method of detecting early lung changes that may be related to metastatic sarcoma tissue in the lung or treatment effects in the lung that are not visually evident or as obvious with visual interpretation of the serial chest CT studies on these patients.

### Evaluation and limitations

We used our previously described QCT method of determining regional lung mechanic using state of the art image registration methods that minimize the cost function of tissue volume difference between the reference and the floating images to provide the optimal local-to-local matching between serial CT scans at similar global lung volumes [9, 12, 16, 18]. It is noted that the cost function does not approach zero in the presence of tissue volumes changes between two different CT studies performed with similar techniques. However, the cost function still helps to minimize the error between matched local image points without exaggerating tissue volume changes.

The adopted SSTVD image similarity metric with a multilevel approach has shown good performances in local to local matching between two volumetric lung CT images even for large deformation [26]. The cost function minimizes the tissue volume changes from larger to smaller regions. This approach provides a conservative condition for any change in regional tissue volumes. However, it does not guarantee the exact quantification of tissue volume changes between the images with addition of a large nodule. In addition, use of different CT scanners, CT protocols and reconstruction algorithms between CT scans of Group 3 sarcoma subjects (**Table 2**) have further effects on image registration accuracy and density-based calculation. However, this is an inevitable condition originating from different clinical CT protocols in this retrospective study. We manually adjusted CT densities that the middle of trachea has -1000 HU and the aorta has 55 HU.

**Fig 7:**
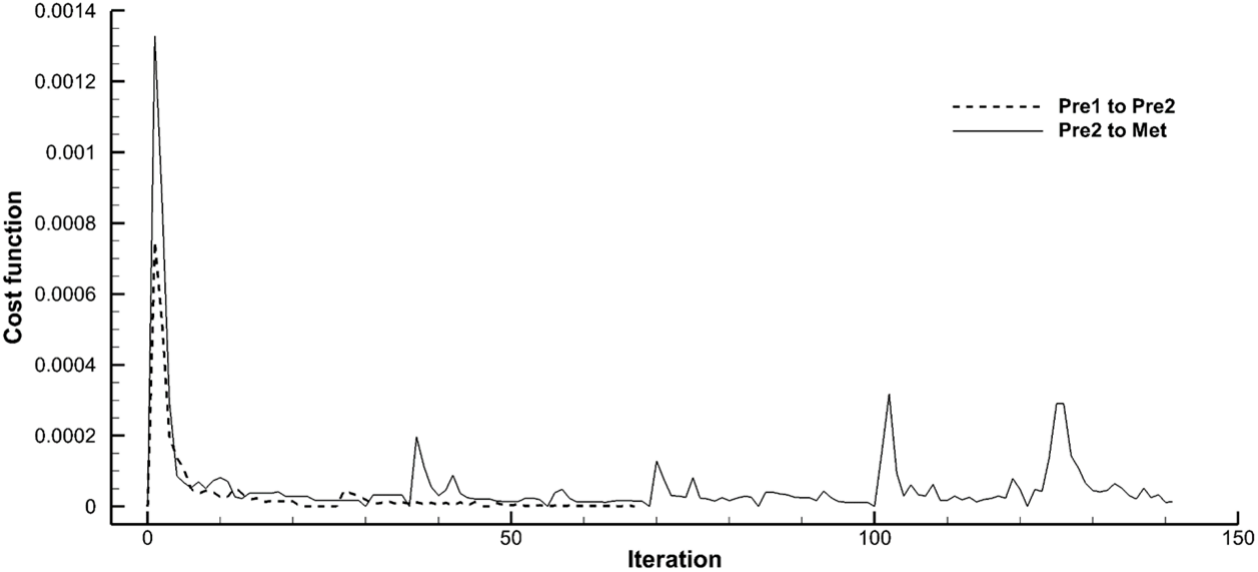
Convergence of cost function during image registration of pre-metastatic and metastatic pairs of a sarcoma patient (Group 3) shown in Fig 4. “Pre1 to Pre2” and “Pre2 to Met” denote for pre-metastatic pair and metastatic pair, respectively.

Despite the limitations, reasonable matching was observed in visual comparison between the reference images and the warped images of the current study (figure not shown). **Fig 7** demonstrates robust convergence of the cost functions of the pre-metastatic scan pairs (Pre1 to Pre2) and the metastatic pair (Pre2 to Met) of the sarcoma subject in **Fig 4**. With occurrence of a large metastatic nodule the iteration required noticeably increased but still ends convergent. Further quantitative evaluations using landmark distances and implementation of a regularization scheme are expected in a future study [27].

Another limitation of this study is the use of a contrast agent for clinical CT image data sets, which do not provide the ideal condition for density-based assessment. We recognize that the presence of contrast agent violates some of the principles of this calculation where we assume only two principle density components to assign percentage air and percentage tissue. The extent to which the presence of contrast can alter the tissue component calculation will vary depending upon the amount of contrast given to an individual subject. However, for all of our analysis, we compare the hyper-expanded regions within the lungs of a given subject to the remaining lung regions. Thus, for any given subject, the effect of the presence of contrast agent will be the same between regions, providing validity to conclusions comparing the two types of lung regions for any given subject even if the absolute value of “tissue” is not quite correct. Exclusion of pulmonary blood vessels in the quantitative analyses of lung parenchyma may help minimize the effect of the contrast agent on the results. The actual tissue fractions in local lung regions with contrast agent may be greater than the computed values.

For Group 1 subjects, 4.13±1.2% of lung regions showed increase in tissue volume denoted by $\Delta_{tissue,50\%}^{*}$ (**Table 3**). The normalized tissue volume change may include possible blood volume changes in the microvasculature between two scans. In addition, this may also indicate that the calculation of $\Delta_{tissue}^{*}$ is relatively sensitive compared to Δ*, which is probably because the tissue regions occupy small volume fractions in the voxel, being sensitive to segmentation of lung, lobes, airways, and vessels. Most of the local lung regions with large deformation in Group 1 are found near the boundaries of the lung.
